# Cardiomyopathy in women with acromegaly: clinical characteristics and associated factors in a Colombian multicenter registry

**DOI:** 10.3389/fendo.2026.1877298

**Published:** 2026-07-09

**Authors:** Santiago Sierra-Castillo, David Aristizabal-Colorado, Andres Arteaga-Arellano, Daniel Miranda-Brazales, Ysamar Aquino, Juan Andres Muñoz-Ordoñez, Daniel Sierra-Castillo, Juan C. Peláez Ortiz, David Alexander Vernaza Trujillo, Juan S. Izquierdo-Condoy, Clara Saldarriaga, Alin Abreu-Lomba

**Affiliations:** 1 Department of Epidemiology, CES University, Medellín, Colombia; 2 Department of Cardiology, University of Antioquia, Medellín, Colombia; 3 Grupo interinstitucional de medicina interna (GIMI 1), Universidad Libre, Cali, Colombia; 4 Department of Internal Medicine, Impulso, Quito, Ecuador; 5 Department of Internal Medicine, International University of Ecuador, Quito, Ecuador; 6 Department of Cardiology, Universidad Abierta Interamericana (UAI), Buenos Aires, Argentina; 7 Faculty of Health Sciences, Icesi University, Cali, Colombia; 8 Faculty of Health Sciences, Universidad Pontificia Bolivariana, Medellín, Colombia; 9 Department of Public Health, Pontificia Universidad Javeriana Cali, Cali, Colombia; 10 Research center, Clínica Imbanaco, Cali, Colombia; 11 One Health Research Group, Universidad de las Américas, Quito, Ecuador; 12 Cardio VID Clinic, Pontificia Bolivariana University, Medellín, Colombia; 13 Department of Endocrinology, Imbanaco Medical Center, Cali, Colombia

**Keywords:** acromegalic heart disease, acromegaly, cardiomyopathy, growth hormone, heart diseases, IGF-1, women

## Abstract

**Background:**

Acromegalic heart disease (AHD) is a common complication of acromegaly; however, evidence specifically focused on women in Latin America remains limited.

**Objective:**

To characterize cardiomyopathy in women with acromegaly and identify clinical factors associated with its presence.

**Methods:**

We conducted a secondary analysis of the multicenter RAPACO-Heart registry in Colombia. Women aged ≥18 years with confirmed acromegaly and available echocardiographic evaluation were included. Clinical, anthropometric, biochemical, and echocardiographic data were analyzed. Cardiomyopathy was defined by echocardiographic abnormalities consistent with AHD. Multivariable logistic regression was used to identify factors independently associated with cardiomyopathy.

**Results:**

A total of 116 women were included, of whom 34 (29.3%) had cardiomyopathy. Women with cardiomyopathy were older and had longer disease duration. Hypertension, arrhythmias, and carpal tunnel syndrome were more frequent in this group. In multivariable analysis, age (adjusted OR 1.07 per year; 95% CI 1.03–1.12; p = 0.001), hypertension (adjusted OR 3.9; 95% CI 1.4–11.1; p = 0.009), arrhythmias (adjusted OR 2.8; 95% CI 1.1–7.4; p = 0.03), and carpal tunnel syndrome (adjusted OR 2.6; 95% CI 1.1–6.5; p = 0.04) were independently associated with cardiomyopathy.

**Conclusions:**

Cardiomyopathy was present in nearly one-third of women with acromegaly and was associated with older age and a higher burden of cardiovascular comorbidities. These findings highlight an underrecognized cardiovascular burden and support targeted cardiovascular assessment in this population.

## 1 Introduction

Acromegalic heart disease (AHD) is among the most frequent comorbidities in patients with acromegaly and encompasses a broad spectrum of cardiovascular abnormalities, including arterial hypertension, accelerated atherosclerosis and coronary artery disease, myocardial hypertrophy, arrhythmias, and left ventricular dysfunction (1–5).

Chronic excess of growth hormone (GH) and insulin-like growth factor 1 (IGF-1) contributes to cardiovascular risk through both direct and indirect mechanisms. At the myocardial level, GH and IGF-1 promote cardiac growth and remodeling, potentially leading to hypertrophy, impaired relaxation, and progressive dysfunction (6). Systemically, acromegaly is associated with metabolic and vascular alterations such as insulin resistance, endothelial dysfunction, and activation of neurohormonal pathways that facilitate the development of arterial hypertension and other cardiovascular comorbidities. Consequently, age, disease duration, and body mass index (BMI) have been consistently linked to a higher likelihood of cardiac involvement in acromegaly (3, 7).

Importantly, the cardiovascular phenotype of acromegaly appears to have evolved over time. Earlier diagnosis improved surgical outcomes, and the broader availability of effective medical therapies have been associated with reports of less frequent or less severe structural cardiac abnormalities in contemporary series compared with historical cohorts (8). Nevertheless, real-world data indicate that AHD remains common: in the Colombian RAPACO-Heart study, approximately one-third of patients with acromegaly had AHD, with left ventricular hypertrophy being the most frequent finding (9).

Regarding sex differences, the median age at diagnosis is relatively consistent across reports, typically occurring in the fifth decade of life, with ranges between 40.5 and 47 years (men: 36.5–48.5 years; women: 38–56 years) (9, 10). In Colombia, the RAPACO-Heart registry reported an AHD prevalence of 31.6%, which was lower in women than in men (9).

However, the overall low prevalence of acromegaly limits the availability of studies specifically focused on characterizing AHD in women. Some reviews have described relevant differences, such as higher body mass index and a greater prevalence of dyslipidemia among female patients (11, 12).

In addition, several studies have reported sex-related variations in cardiac parameters, leading to the hypothesis that gender-related differences may influence the development of cardiac abnormalities in this population, potentially mediated by BMI, smoking status, IGF-1 levels, and structural cardiac changes (12, 13).

In this context, the present study aims to characterize the presentation of AHD in women, describe the most frequent comorbidities, and identify factors associated with its presence.

## 2 Materials and methods

### 2.1 Study design and setting

**Figure 1:**
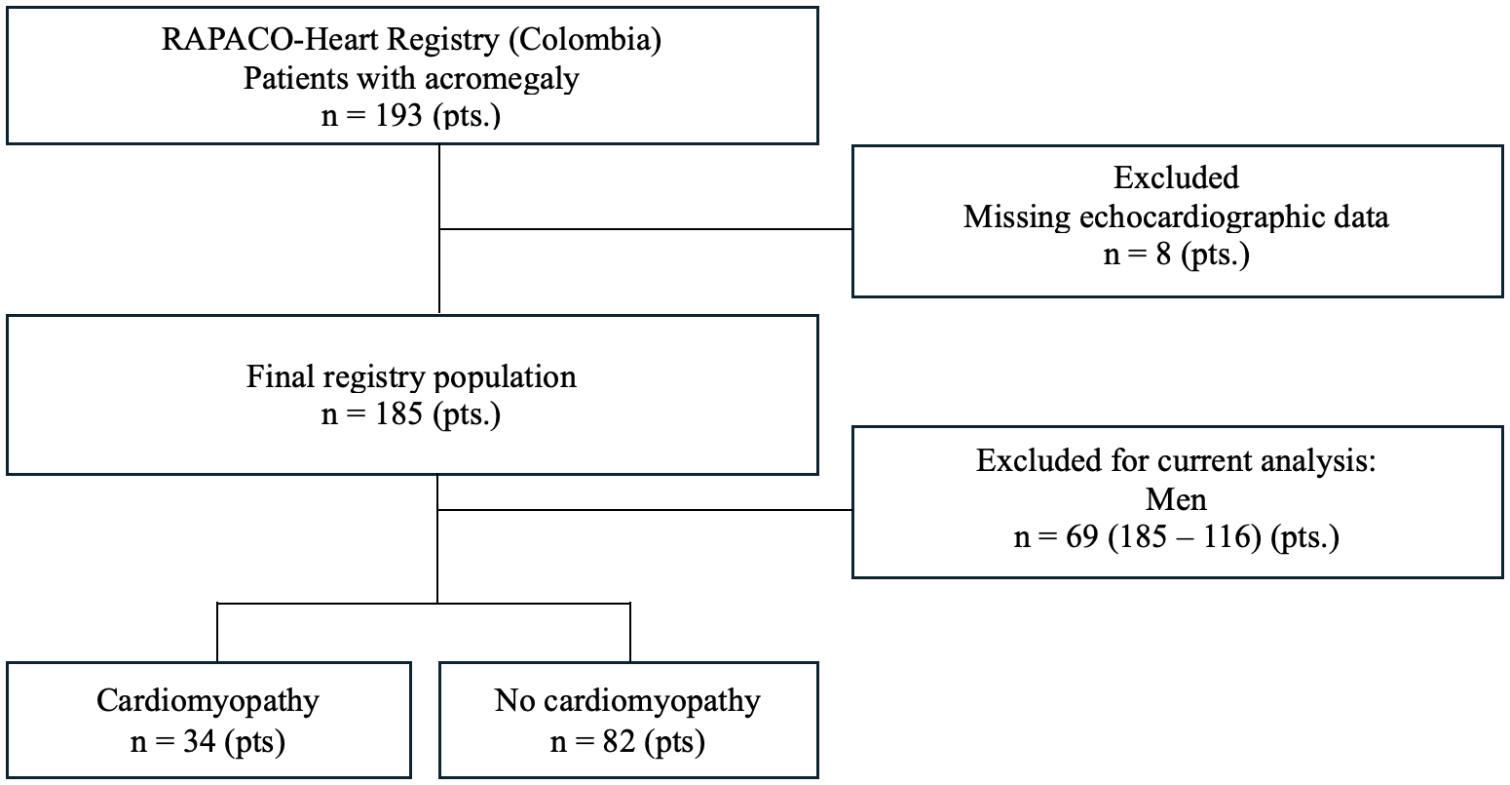
Patient selection flowchart for the study of cardiomyopathy in women with acromegaly. Flowchart illustrating the selection of patients from the RAPACO-Heart registry. A total of 193 patients with acromegaly were initially identified. Patients with missing echocardiographic data (n = 8) were excluded, resulting in a final registry population of 185 patients. Men (n = 69) were excluded for the present analysis. The final analytical sample included 116 women, of whom 34 had cardiomyopathy and 82 did not.

This study was a secondary analysis of the RAPACO-Heart registry (9), a multicenter observational cohort designed to evaluate cardiovascular disease in patients with acromegaly in Colombia. The present study focused exclusively on women with acromegaly (**Figure 1**).

### 2.2 Study population

Women aged ≥18 years with a confirmed diagnosis of acromegaly were eligible for inclusion. Acromegaly was defined by elevated age-adjusted insulin-like growth factor 1 (IGF-1) levels, lack of growth hormone (GH) suppression during oral glucose tolerance testing, and radiological evidence of a pituitary adenoma. Patients with incomplete cardiovascular evaluation or missing essential variables were excluded.

### 2.3 Data collection

Data were obtained from the RAPACO-Heart registry, in which clinical information is recorded as part of routine care. Demographic, clinical, anthropometric, biochemical, and echocardiographic variables were collected from medical records by trained investigators at each participating center.

Echocardiographic findings were reported according to routine clinical practice and documented by the treating physicians. No centralized image analysis or core laboratory adjudication was performed.

### 2.4 Data sources and measurements

Clinical, anthropometric, biochemical, hormonal, imaging, and treatment-related data were retrospectively extracted from standardized electronic case report forms used across participating centers. Collected clinical and anthropometric variables included age, disease duration, weight, height, body mass index (BMI), neck circumference, abdominal circumference, and hip circumference. Information on comorbidities was also recorded, including hypertension, diabetes mellitus, prediabetes, arrhythmias, sleep apnea severity, hypopituitarism, carpal tunnel syndrome, osteopenia, osteoporosis, fatty liver disease, cholelithiasis, and gallbladder polyps.

Biochemical and hormonal variables included baseline and follow-up GH and IGF-1 levels, prolactin, fasting glucose, and tumor proliferation markers such as Ki-67 and p53. To account for age-related variability in reference ranges, IGF-1 concentrations were additionally normalized to the age-adjusted upper limit of normal (ULN) and expressed as multiples of the ULN (×ULN) (14, 15).

### 2.5 Outcome definition

Cardiac evaluation was performed using transthoracic echocardiography according to local institutional acquisition protocols. To ensure standardized outcome classification, echocardiographic definitions of cardiac structural and functional abnormalities were derived from the recommendations of the American Society of Echocardiography (ASE) for cardiac chamber quantification and ventricular function (16). Accordingly, the primary outcome—cardiomyopathy—was operationally defined as the presence of guideline-based echocardiographic abnormalities consistent with acromegalic heart disease, including left ventricular hypertrophy, biventricular hypertrophy, systolic dysfunction, or structural cardiac remodeling according to ASE criteria (17–20). Participants were categorized according to the presence or absence of cardiomyopathy.

Isolated valvular disease without evidence of myocardial involvement was not considered cardiomyopathy. Diastolic dysfunction alone was not used as a standalone criterion unless it was accompanied by structural myocardial abnormalities compatible with acromegalic heart disease.

### 2.6 Bias and study size

Because this was a secondary analysis of a multicenter registry, efforts to reduce information bias included the use of standardized electronic case report forms across participating centers. Selection bias was minimized by including all eligible women in the registry who met the predefined inclusion criteria. In addition, multivariable regression analysis was performed to reduce the effect of potential confounding when evaluating factors associated with cardiomyopathy.

The study size was determined by the number of eligible women with acromegaly available in the RAPACO-Heart registry who fulfilled the inclusion criteria during the study period.

### 2.7 Statistical analysis

Continuous variables were summarized as mean ± standard deviation or median with interquartile range, according to their distribution, whereas categorical variables were presented as frequencies and percentages. Before inferential analyses, numerical variables were assessed for distribution using the Kolmogorov–Smirnov test to guide the selection of summary measures and comparative tests. Variables with more than 15% missing data were excluded from inferential analyses. Missing continuous values were handled using simple mean imputation, whereas observations with missing categorical data were excluded from the corresponding analyses.

Comparisons between women with and without cardiomyopathy were performed using Student’s t test for normally distributed continuous variables and the Mann–Whitney U test for non-normally distributed variables. Categorical variables were compared using the chi-square test or Fisher’s exact test, as appropriate, with Fisher’s exact test preferred when expected cell counts were small. Exploratory univariable analyses were first performed to evaluate crude associations between candidate variables and cardiomyopathy.

To identify factors independently associated with cardiomyopathy, multivariable logistic regression models were constructed. Candidate variables were selected based on clinical relevance and/or an association with cardiomyopathy in univariable analysis at a p value <0.25. Age was included as an *a priori* confounder. Forward stepwise selection was used for final model construction. The final multivariable model included age, hypertension, arrhythmias, and carpal tunnel syndrome. Continuous variables were assessed for linearity with the log-odds of the outcome, and multicollinearity among predictors was evaluated using variance inflation factor (VIF) analysis. Model calibration was assessed using the Hosmer–Lemeshow goodness-of-fit test. Given the limited number of cardiomyopathy events, model complexity was intentionally restricted to reduce the risk of overfitting. Interaction terms were not included because of the exploratory nature of the study and the limited number of events.

Adjusted odds ratios (aORs) with 95% confidence intervals (CIs) were reported. A two-sided p value <0.05 was considered statistically significant. All statistical analyses were performed using Jamovi version 2.7.24, an R-based statistical software platform.

### 2.8 Ethical considerations

The RAPACO-Heart registry was reviewed and approved by the Institutional Review Board of Clínica Imbanaco, Colombia, under approval code CEI-878. The study was conducted in accordance with the principles of the Declaration of Helsinki. This study involved human participant registry data collected retrospectively as part of routine clinical care. For the present secondary analysis, only anonymized data were used. Therefore, the requirement for additional informed consent was waived by the corresponding ethics committee in accordance with institutional regulations and the retrospective observational nature of the study.

## 3 Results

### 3.1 Study population and clinical characteristics

A total of 116 women with acromegaly were included in the analysis, of whom 34 (29.3%) had cardiomyopathy and 82 (70.7%) did not (**Figure 1**).

Women with cardiomyopathy were significantly older than those without cardiomyopathy (60.1 ± 12.4 vs. 47.5 ± 13.9 years; p < 0.001). Disease duration was also longer in the cardiomyopathy group (7.5 [5.0–11.8] vs. 6.0 [4.0–7.8] years; p = 0.012). In contrast, body weight, height, body mass index, and anthropometric measures relevant to acromegaly, including neck, abdominal, and hip circumferences, were similar between groups (all p > 0.05) (**Table 1**).

**Table 1:** Clinical and anthropometric characteristics of women with acromegaly according to cardiomyopathy status.

Variable	Cardiomyopathy (n = 34)	No cardiomyopathy (n = 82)	p value
Age, years (mean ± SD)	60.1 ± 12.4	47.5 ± 13.9	<0.001
Weight, kg (mean ± SD)	72.8 ± 14.6	75.1 ± 15.2	0.45
Height, m (mean ± SD)	1.59 ± 0.06	1.60 ± 0.07	0.62
BMI, kg/m² (mean ± SD)	27.4 ± 5.1	28.1 ± 6.2	0.53
Neck circumference, cm (median [IQR])	40 [38–43]	39 [37–42]	0.29
Abdominal circumference, cm (median [IQR])	103 [95–115]	98 [90–110]	0.18
Hip circumference, cm (median [IQR])	108 [102–118]	106 [98–116]	0.41
Disease duration, years (median [IQR])	7.5 [5.0–11.8]	6.0 [4.0–7.8]	0.012

Continuous variables are presented as mean ± SD or median (IQR). Group comparisons were performed using Student’s t-test or Mann–Whitney U test, as appropriate. p < 0.05 was considered statistically significant. BMI, body mass index; SD, standard deviation; IQR, interquartile range.

### 3.2 Comorbidities

Hypertension was significantly more prevalent among women with cardiomyopathy than among those without cardiomyopathy (82.4% vs. 47.6%; p = 0.002). Arrhythmias (32.4% vs. 12.2%; p = 0.01) and carpal tunnel syndrome (55.9% vs. 29.3%; p = 0.01) were also more frequent in the cardiomyopathy group (**Table 2**).

**Table 2:** Comorbidities in women with acromegaly according to cardiomyopathy status.

Variable	Cardiomyopathy (n = 34)	No cardiomyopathy (n = 82)	p value
Hypopituitarism	11 (32.4%)	15 (18.3%)	0.180
Diabetes mellitus	10 (29.4%)	14 (17.1%)	0.140
Prediabetes	2 (5.9%)	1 (1.2%)	0.205
Hypertension	28 (82.4%)	39 (47.6%)	0.002
Arrhythmias	11 (32.4%)	10 (12.2%)	0.010
Mild sleep apnea	7 (20.6%)	11 (13.4%)	0.720
Moderate sleep apnea	5 (14.7%)	13 (15.9%)	0.580
Severe sleep apnea	6 (17.6%)	9 (11.0%)	0.610
Carpal tunnel syndrome	19 (55.9%)	24 (29.3%)	0.010
Osteopenia	16 (47.1%)	23 (28.0%)	0.070
Osteoporosis	6 (17.6%)	8 (9.8%)	0.260
Fatty liver disease	13 (38.2%)	25 (30.5%)	0.440
Cholelithiasis	9 (26.5%)	15 (18.3%)	0.350
Gallbladder polyp	2 (5.9%)	3 (3.7%)	0.610

Categorical variables are presented as frequencies and percentages. Comparisons between groups were performed using the chi-square test or Fisher’s exact test, as appropriate. p < 0.05 was considered statistically significant.

**Figure 2:**
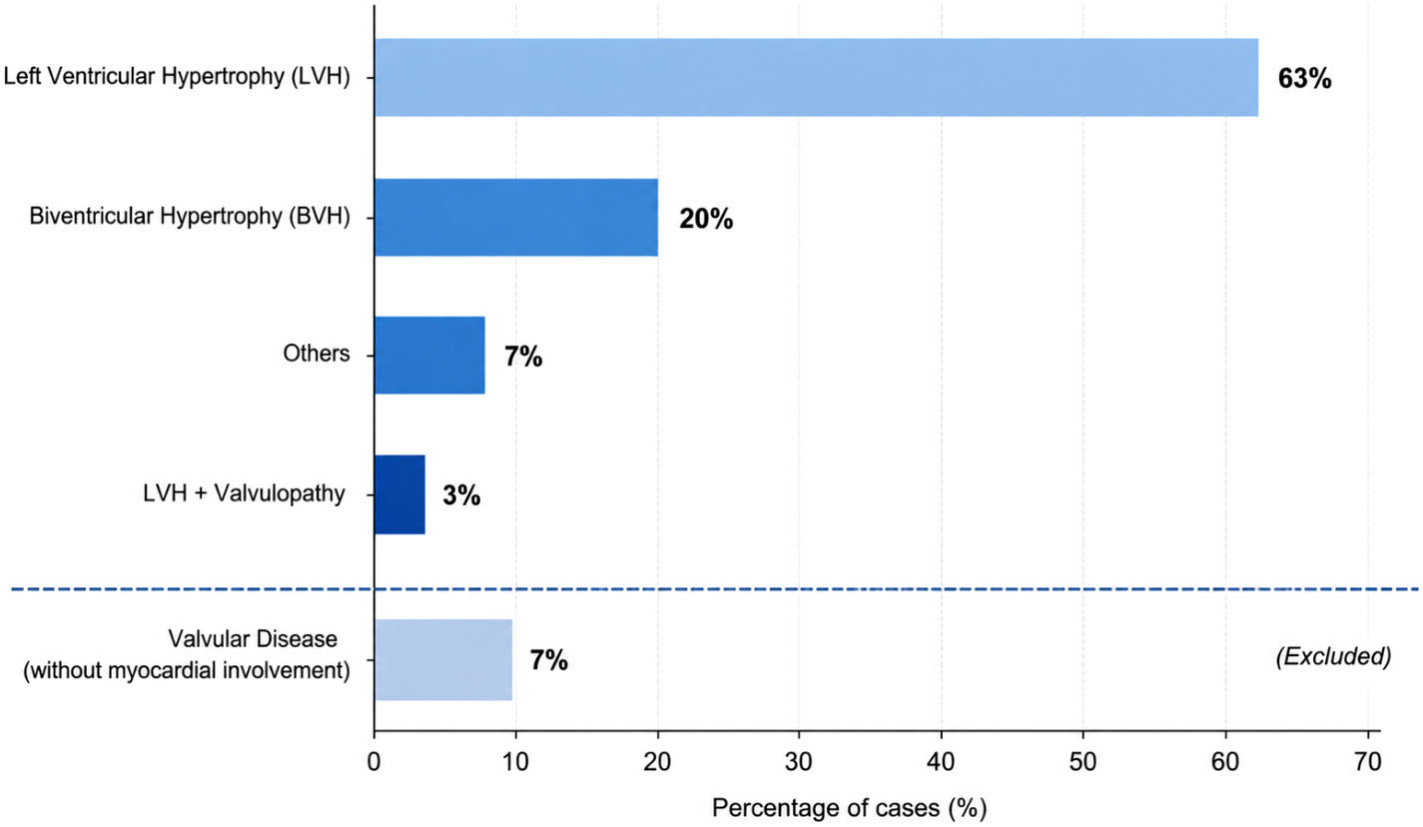
Distribution of echocardiographic abnormalities among women classified as having cardiomyopathy. Left ventricular hypertrophy was the most frequent finding, followed by biventricular hypertrophy, other structural abnormalities, and left ventricular hypertrophy associated with valvular disease. Values are expressed as percentages of the echocardiographic categories displayed in the figure. Isolated valvular disease without myocardial involvement is shown separately as an excluded category and was not included in the cardiomyopathy definition.

Among women classified as having cardiomyopathy, left ventricular hypertrophy was the most frequent echocardiographic abnormality, followed by biventricular hypertrophy, other structural abnormalities, and left ventricular hypertrophy associated with valvular disease. Isolated valvular disease without myocardial involvement was not considered cardiomyopathy and was shown separately as an excluded category in **Figure 2**.

No significant between-group differences were observed for hypopituitarism, diabetes mellitus, prediabetes, sleep apnea severity, osteoporosis, fatty liver disease, cholelithiasis, or gallbladder polyps. Osteopenia was more common among women with cardiomyopathy, although the difference did not reach statistical significance (47.1% vs. 28.0%; p = 0.07).

### 3.3 Hormonal and biochemical profile

Baseline IGF-1 and GH concentrations tended to be higher in women with cardiomyopathy, although these differences were not statistically significant (IGF-1: 690 [520–890] vs. 560 [410–720] ng/mL, p = 0.09; GH: 12.5 [6.8–21.4] vs. 8.1 [3.9–15.2] ng/mL, p = 0.12). Similarly, IGF-1 expressed as multiples of the upper limit of normal was numerically higher in the cardiomyopathy group (3.7 [2.8–4.8] vs. 2.9 [2.1–3.7]; p = 0.09) (**Table 3**). Follow-up IGF-1 and GH levels, Ki-67 and p53 indices, fasting glucose, and prolactin concentrations did not differ significantly between groups.

**Table 3:** Hormonal and biochemical variables according to cardiomyopathy status.

Variable	Cardiomyopathy (n = 34)	No cardiomyopathy (n = 82)	p value
Baseline IGF-1 (ng/mL)	690 [520–890]	560 [410–720]	0.09
Baseline GH (ng/mL)	12.5 [6.8–21.4]	8.1 [3.9–15.2]	0.12
IGF-1 at follow-up (ng/mL)	310 [220–410]	295 [210–390]	0.64
IGF-1 (×ULN), median [IQR]	3.7 [2.8–4.8]	2.9 [2.1–3.7]	0.09
GH at follow-up (ng/mL)	2.8 [1.2–6.5]	2.4 [1.0–5.9]	0.71
Ki-67 (%)	2.0 [1.0–3.5]	1.5 [1.0–2.5]	0.15
p53 (%)	10 [5–20]	8 [5–15]	0.21
Fasting glucose (mg/dL)	102 [94–118]	99 [92–112]	0.48
Prolactin (ng/mL)	18.5 [12.4–28.6]	17.2 [11.1–26.3]	0.67

Continuous variables are presented as median (IQR). Comparisons between groups were performed using the Mann–Whitney U test. p < 0.05 was considered statistically significant.

IGF-1, insulin-like growth factor 1; GH, growth hormone; ULN, upper limit of normal; IQR, interquartile range.

### 3.4 Factors independently associated with cardiomyopathy

In the multivariable logistic regression analysis, increasing age was independently associated with the presence of cardiomyopathy (adjusted odds ratio [OR] 1.07 per year; 95% confidence interval [CI] 1.03–1.12; p = 0.001). Hypertension (adjusted OR 3.9; 95% CI 1.4–11.1; p = 0.009), arrhythmias (adjusted OR 2.8; 95% CI 1.1–7.4; p = 0.03), and carpal tunnel syndrome (adjusted OR 2.6; 95% CI 1.1–6.5; p = 0.04) also remained independently associated with cardiomyopathy (**Table 4**).

**Table 4:** Multivariable logistic regression model for factors associated with cardiomyopathy.

Variable	Adjusted OR	95% CI	p value
Age (per year)	1.07	1.03–1.12	0.001
Hypertension	3.9	1.4–11.1	0.009
Arrhythmias	2.8	1.1–7.4	0.030
Carpal tunnel syndrome	2.6	1.1–6.5	0.040

Results are presented as adjusted OR with 95% confidence intervals (CI). A multivariable logistic regression model was used to identify factors independently associated with cardiomyopathy. p < 0.05 was considered statistically significant.

OR, odds ratio; CI, confidence interval.

## 4 Discussion

This study identified several factors associated with cardiomyopathy in women with acromegaly. Older age, longer disease duration, hypertension, arrhythmias, and carpal tunnel syndrome were associated with a higher likelihood of cardiomyopathy. These findings are consistent with previous evidence indicating that cardiovascular involvement in acromegaly is strongly influenced by disease duration and cumulative exposure to GH and IGF-1 excess, both of which tend to increase with age and contribute to progressive myocardial remodeling (17, 21, 22). In our cohort, the proportion of women with cardiomyopathy was 29.3% (95% confidence interval 21.0%–37.6%), highlighting the substantial burden of cardiac involvement in this population.

Cardiomyopathy was operationally defined as the presence of guideline-based echocardiographic structural or functional abnormalities compatible with acromegalic heart disease. We acknowledge that some findings, particularly left ventricular hypertrophy and concentric remodeling, may overlap with hypertensive heart disease. However, left ventricular hypertrophy is a cardinal manifestation of acromegalic cardiac involvement and may occur even in non-hypertensive patients or in the absence of other traditional cardiovascular risk factors. Moreover, hypertension in acromegaly should not always be interpreted as an unrelated comorbidity, since it may represent a secondary, disease-related manifestation of chronic GH/IGF-1 excess and its associated cardiovascular effects. Therefore, given the difficulty of reliably distinguishing isolated hypertensive remodeling from acromegaly-related myocardial remodeling, hypertension was not considered an exclusion criterion for acromegalic heart disease. Instead, echocardiographic abnormalities were interpreted within the broader clinical context of acromegaly and classified using standardized ASE-based criteria (5, 23, 24).

Age was associated with the presence of cardiomyopathy in our cohort. This observation is consistent with large observational studies and registries showing that patients with cardiovascular complications at diagnosis are significantly older than those without such involvement (11, 12). Prolonged exposure to GH and IGF-1 excess has been associated with myocardial hypertrophy, interstitial remodeling, and eventual functional impairment, supporting the interpretation of age as a surrogate marker of cumulative hormonal burden rather than an isolated risk factor (21, 25). In women, increasing age is also closely linked to menopausal status, which may further contribute to cardiovascular risk through well-established metabolic and hormonal changes.

Hypertension and arrhythmias were more frequent among women with cardiomyopathy and remained independently associated with cardiomyopathy in the multivariable model. Hypertension is one of the most prevalent cardiovascular comorbidities in acromegaly and has been closely linked to the severity of acromegalic cardiomyopathy and left ventricular hypertrophy (12, 26). Similarly, arrhythmias have been described as clinical manifestations of advanced myocardial involvement, reflecting structural remodeling and electrical instability associated with chronic GH/IGF-1 exposure (19).

The high prevalence of hypertension in our cohort warrants careful interpretation of these findings, as hypertension is a well-established driver of myocardial hypertrophy and cardiac remodeling and may therefore contribute to the structural cardiac changes observed in this population (27–30). However, these alterations likely reflect mixed mechanisms involving both chronic GH/IGF-1 exposure and coexisting cardiovascular comorbidities, particularly hypertension (17, 19, 20, 31). In this context, hypertension may act both as a confounder and as a synergistic factor that amplifies myocardial remodeling in acromegaly.

Consistent with this, previous echocardiographic studies have shown that patients with acromegaly frequently present with increased left ventricular mass, wall thickness, and left atrial volume, along with impaired diastolic function despite preserved systolic function, and that some of these abnormalities may be partially attributable to coexisting conditions such as hypertension, metabolic disorders, and demographic factors rather than being exclusively driven by GH/IGF-1 excess. This overlap has been widely described and represents a relevant challenge when interpreting cardiovascular involvement in patients with acromegaly (17, 19, 20, 28).

In the broader RAPACO-Heart cohort that included both men and women, some cardiovascular associations were attenuated after multivariable adjustment, suggesting the influence of confounding factors such as age and other clinical variables (9). In contrast, in the present women-only analysis, hypertension and arrhythmias remainedindependently associated with cardiomyopathy, supporting the relevance of these variables in the female acromegaly population. Despite this, the observed trends are consistent with previous evidence reporting a higher prevalence of hypertension in women with acromegaly than in men, supporting the hypothesis that sex-related biological factors may contribute to cardiovascular involvement in this population (26, 32). In this context, previous studies have suggested that women with acromegaly may exhibit a greater degree of myocardial fibrosis, which could contribute to cardiomyopathy and conduction abnormalities. These observations may help explain the coexistence of hypertension, arrhythmias, and cardiac remodeling observed in our cohort and support the need for further investigation of potential sex-related differences in cardiovascular involvement (10, 17, 20, 21, 28, 32–37).

Additionally, the interaction between sex and age may contribute to cardiovascular involvement in women with acromegaly, as older women may experience longer cumulative exposure to GH and IGF-1 excess, potentially increasing susceptibility to myocardial remodeling and cardiovascular complications (17). This interaction provides a biologically plausible explanation for the associations observed in our cohort (26, 32). Furthermore, aging in women is associated with menopause-related metabolic changes, including increased visceral adiposity, insulin resistance, and metabolic syndrome (22, 26). These alterations may be particularly relevant in women with acromegaly, in whom greater insulin resistance and compensatory hyperinsulinemia have been described despite similar glucose levels (21, 32). In this context, the coexistence of chronic GH/IGF-1 excess and menopause-related metabolic changes may synergistically contribute to cardiovascular risk and cardiac remodeling (20, 31, 37).

Experimental and imaging-based studies have shown that chronic exposure to excess GH and IGF-1 promotes myocardial hypertrophy, interstitial remodeling, and electrical instability, mechanisms that may contribute to the development of acromegalic heart disease and rhythm disturbances in advanced stages of the disease (17, 21, 25, 31, 38, 39).

Carpal tunnel syndrome also emerged as an independent factor associated with cardiomyopathy. This finding is clinically relevant, as carpal tunnel syndrome is a frequent manifestation of acromegaly and has been associated with greater systemic involvement and longer disease exposure. Data from the Colombian RAPACO registry, including analyses not primarily focused on cardiovascular outcomes (9), have shown a high prevalence of musculoskeletal and metabolic comorbidities in both men and women, supporting the interpretation of carpal tunnel syndrome as a marker of broader disease burden and chronic exposure to GH/IGF-1 excess rather than a direct causal factor in cardiomyopathy (21).

Baseline GH and IGF-1 levels were numerically higher among women with cardiomyopathy, although these differences did not reach statistical significance. This lack of statistical significance may be explained by heterogeneity in disease duration, prior treatment exposure, and the limitations of single time-point hormonal measurements, which may not accurately reflect cumulative hormonal exposure over time. In our cohort, women with cardiomyopathy also had a longer disease duration, further supporting the role of prolonged exposure to GH and IGF-1 excess in the development of cardiac involvement. Previous studies have emphasized that longitudinal biochemical control plays a key role in modulating cardiac remodeling and improving cardiovascular outcomes in patients with acromegaly (31). Accordingly, the absence of statistically significant hormonal differences in our study may reflect limited statistical power due to the relatively small sample size, which is an expected challenge in studies of rare diseases such as acromegaly. Therefore, these hormonal findings should be considered exploratory and hypothesis-generating rather than confirmatory.

To the best of our knowledge, this is the first study specifically addressing cardiomyopathy in women with acromegaly in Colombia. By focusing on a female population derived from a national multicenter registry, this study provides novel regional data on cardiovascular involvement among women with acromegaly, an underrepresented population in the literature (40). Our findings suggest that cardiomyopathy in women with acromegaly is closely linked to older age and a higher burden of cardiovascular and systemic comorbidities, particularly hypertension, arrhythmias, and carpal tunnel syndrome. These results support the need for targeted cardiovascular assessment and follow-up in women with acromegaly, especially in those presenting with these clinical features, and underscore the importance of early diagnosis and comprehensive management to mitigate long-term cardiac complications.

## 5 Conclusions

In this cohort of women with acromegaly, cardiomyopathy was common and was associated with older age and a greater burden of cardiovascular comorbidities, particularly hypertension and arrhythmias, highlighting a clinically relevant but often underrecognized cardiovascular burden in this population. Carpal tunnel syndrome emerged as a marker of broader systemic involvement and likely longer disease exposure, supporting its role as an indicator of advanced disease rather than an isolated comorbidity. These findings underscore the importance of targeted cardiovascular assessment and follow-up in women with acromegaly, especially in those presenting with hypertension, arrhythmias, or carpal tunnel syndrome, as well as the need for early diagnosis and comprehensive management to reduce long-term cardiac complications.

## 6 Strengths and limitations

This study has several strengths. It is based on a multicenter national registry, providing real-world data that enhance external validity and generalizability. By focusing specifically on women with acromegaly, an underrepresented population in the literature, this study addresses an important knowledge gap and provides novel insights into cardiovascular involvement in a women-only acromegaly cohort. In addition, the comprehensive assessment of clinical, anthropometric, and biochemical variables, together with the use of multivariable regression analysis, strengthens the identification of factors independently associated with cardiomyopathy.

The study also has limitations. Its observational and cross-sectional design precludes causal inference and limits the assessment of temporal relationships. Because the study included only women, direct comparisons with men could not be performed, and no definitive conclusions can be drawn regarding sex-specific susceptibility to cardiomyopathy. Associations of hypertension and arrhythmias with cardiomyopathy may have been influenced by residual confounding, particularly by age and the limited sample size, which is an expected challenge in studies of rare diseases such as acromegaly. Furthermore, detailed echocardiographic parameters, staging of cardiac involvement, and standardized diagnostic thresholds were not systematically available for all patients, as cardiac findings were derived from routine clinical reports rather than centralized image analysis. This limitation may have introduced heterogeneity in the classification of cardiac involvement and limited differentiation between acromegalic and hypertensive cardiac remodeling. Finally, single time-point GH and IGF-1 measurements may have limited the assessment of cumulative hormonal exposure and progressive cardiac remodeling.

## Data Availability

The original contributions presented in the study are included in the article/supplementary material. Further inquiries can be directed to the corresponding author.

## References

[1] Hong S Kim K-S Han K Park C-Y . Acromegaly and cardiovascular outcomes: a cohort study. Eur Heart J. (2022) 43:1491–9. doi: 10.1093/eurheartj/ehab822 34864952

[2] Wolters TLC Netea MG Riksen NP Hermus ARMM Netea-Maier RT . Acromegaly, inflammation and cardiovascular disease: a review. Rev Endocr Metab Disord. (2020) 21:547–68. doi: 10.1007/s11154-020-09560-x 32458292 PMC7560935

[3] McGuffin WL Sherman BM Roth F Gorden P Kahn CR Roberts WC . Acromegaly and cardiovascular disorders. Ann Intern Med. (1974) 81:11–8. doi: 10.7326/0003-4819-81-1-11 4276173

[4] Sharma MD Nguyen AV Brown S Robbins RJ . Cardiovascular disease in acromegaly. Methodist DeBakey Cardiovasc J. (2017) 13:64–7. doi: 10.14797/mdcj-13-2-64 28740584 PMC5512681

[5] Giustina A Colao A . Acromegaly. N Engl J Med. (2025) 393:1926–39. doi: 10.1056/NEJMra2409076 41223366

[6] Dewanjee A Rayes D Tehrani B Shah P Howard E Kennedy J . An uncommon cause of heart failure: acromegalic cardiomyopathy culminating in orthotopic heart transplantation. US Cardiol Rev. (2026) 20:2. doi: 10.15420/usc.2026.20.s1

[7] Abreu Lomba A Corredor-Rengifo D Mejia Velez CA Carvajal Ortiz R Pantoja Guerrero D Arenas HM . Biochemical control in a Colombian cohort of patients with acromegaly: a 12-month follow-up study (2017-2023). Cureus. (2024) 16:e75553. doi: 10.7759/cureus.75553 39803157 PMC11724446

[8] Lavrentaki A Paluzzi A Wass JAH Karavitaki N . Epidemiology of acromegaly: review of population studies. Pituitary. (2017) 20:4–9. doi: 10.1007/s11102-016-0754-x 27743174 PMC5334410

[9] Abreu-Lomba A Aristizábal-Colorado D Sierra-Castillo S Weir-Restrepo D Gómez-Mercado CA Vernaza Trujillo DA . Prevalence and characteristics of heart disease in patients with acromegaly in Colombia (RAPACO HEART Study). Cardiology. (2025) 151:226–35. doi: 10.1159/000545875 40349690

[10] Gatto F Arecco A De Alcubierre D De Simone M Gentiluomo R Lanzi V . Gender differences in the glycometabolic and cardiovascular features of acromegaly. Pituitary. (2026) 29:34. doi: 10.1007/s11102-025-01629-7 41569446

[11] Mestrón A Webb SM Astorga R Benito P Catalá M Gaztambide S . Epidemiology, clinical characteristics, outcome, morbidity and mortality in acromegaly based on the Spanish Acromegaly Registry (Registro Espanol de Acromegalia, REA). Eur J Endocrinol. (2004) 151:439–46. doi: 10.1530/eje.0.1510439 15476442

[12] Petrossians P Daly AF Natchev E Maione L Blijdorp K Sahnoun-Fathallah M . Acromegaly at diagnosis in 3173 patients from the Liège Acromegaly Survey (LAS) Database. (2017) 24(10):505–518. doi: 10.1530/ERC-17-0253 PMC5574208 28733467

[13] Guo X Cao J Liu P Cao Y Li X Gao L . Cardiac abnormalities in acromegaly patients: a cardiac magnetic resonance study. Int J Endocrinol. (2020) 2020:2018464. doi: 10.1155/2020/2018464 32148485 PMC7042537

[14] Melmed S di Filippo L Fleseriu M Mercado M Karavitaki N Gurnell M . Consensus on acromegaly therapeutic outcomes: an update. Nat Rev Endocrinol. (2025) 21:718–37. doi: 10.1038/s41574-025-01148-2 40804505

[15] Giustina A Biermasz N Casanueva FF Fleseriu M Mortini P Strasburger C . Consensus on criteria for acromegaly diagnosis and remission. Pituitary. (2024) 27:7–22. doi: 10.1007/s11102-023-01360-1 37923946 PMC10837217

[16] Lang RM Badano LP Mor-Avi V Afilalo J Armstrong A Ernande L . Recommendations for cardiac chamber quantification by echocardiography in adults: an update from the American Society of Echocardiography and the European Association of Cardiovascular Imaging. J Am Soc Echocardiogr Off Publ Am Soc Echocardiogr. (2015) 28:1–39.e14. doi: 10.1016/j.echo.2014.10.003 25559473

[17] Wolf P Maione L Kamenický P Chanson P . Acromegalic cardiomyopathy: an entity on its own? The effects of GH and IGF-I excess and treatment on cardiovascular risk factors. Arch Med Res. (2023) 54:102921. doi: 10.1016/j.arcmed.2023.102921 38040526

[18] Iglesias P . Acromegaly and cardiovascular disease: associated cardiovascular risk factors, cardiovascular prognosis, and therapeutic impact. J Clin Med. (2025) 14:1–19. doi: 10.3390/jcm14061906 40142714 PMC11943432

[19] Sharma AN Tan M Amsterdam EA Singh GD . Acromegalic cardiomyopathy: epidemiology, diagnosis, and management. Clin Cardiol. (2018) 41:419–25. doi: 10.1002/clc.22867 29574794 PMC6489905

[20] Popielarz-Grygalewicz A Gąsior JS Konwicka A Grygalewicz P Stelmachowska-Banaś M Zgliczyński W . Heart in acromegaly: the echocardiographic characteristics of patients diagnosed with acromegaly in various stages of the disease. Int J Endocrinol. (2018) 2018:6935054. doi: 10.1155/2018/6935054 30123265 PMC6079421

[21] Saccà L Cittadini A Fazio S . Growth hormone and the heart. Endocr Rev. (1994) 15:555–73. doi: 10.1210/edrv-15-5-555 7843068

[22] Colao A Grasso LFS Di Cera M Thompson-Leduc P Cheng WY Cheung HC . Association between biochemical control and comorbidities in patients with acromegaly: an Italian longitudinal retrospective chart review study. J Endocrinol Invest. (2020) 43:529–38. doi: 10.1007/s40618-019-01138-y 31741320 PMC7067716

[23] Januszewicz A Mulatero P Dobrowolski P Monticone S Van der Niepen P Sarafidis P . Cardiac phenotypes in secondary hypertension: JACC state-of-the-art review. J Am Coll Cardiol. (2022) 80:1480–97. doi: 10.1016/j.jacc.2022.08.714 36202538

[24] Fleseriu M Langlois F Lim DST Varlamov EV Melmed S . Acromegaly: pathogenesis, diagnosis, and management. Lancet Diabetes Endocrinol. (2022) 10:804–26. doi: 10.1016/S2213-8587(22)00244-3 36209758

[25] Clayton RN . Cardiovascular function in acromegaly. Endocr Rev. (2003) 24:272–7. doi: 10.1210/er.2003-0009 12788799

[26] Ramos-Leví AM Marazuela M . Bringing cardiovascular comorbidities in acromegaly to an update. How should we diagnose and manage them? Front Endocrinol. (2019) 10. doi: 10.3389/fendo.2019.00120 30930848 PMC6423916

[27] Tomek J Bub G . Hypertension-induced remodelling: on the interactions of cardiac risk factors. J Physiol. (2017) 595:4027–36. doi: 10.1113/JP273043 28217927 PMC5471416

[28] Yildiz M Oktay AA Stewart MH Milani RV Ventura HO Lavie CJ . Left ventricular hypertrophy and hypertension. Prog Cardiovasc Dis. (2020) 63:10–21. doi: 10.1016/j.pcad.2019.11.009 31759953

[29] González A Ravassa S López B Moreno MU Beaumont J San José G . Myocardial remodeling in hypertension. Hypertension. (2018) 72:549–58. doi: 10.1161/HYPERTENSIONAHA.118.11125 30354762

[30] Wang BX . Diagnosis and management of hypertensive heart disease: incorporating 2023 European Society of Hypertension and 2024 European Society of Cardiology guideline updates. J Cardiovasc Dev Dis. (2025) 12:12–46. doi: 10.3390/jcdd12020046 39997480 PMC11856785

[31] Anderson LJ Tamayose JM Garcia JM . Use of growth hormone, IGF-I, and insulin for anabolic purpose: pharmacological basis, methods of detection, and adverse effects. Mol Cell Endocrinol. (2018) 464:65–74. doi: 10.1016/j.mce.2017.06.010 28606865 PMC5723243

[32] Sherin RPV Vietor NO Usman A Hoang TD Shakir MKM . Cardiovascular disorders associated with acromegaly: an update. Endocr Pract Off J Am Coll Endocrinol Am Assoc Clin Endocrinol. (2024) 30:1212–9. doi: 10.1016/j.eprac.2024.09.014 39332498

[33] Kannel WB Hjortland MC McNamara PM Gordon T . Menopause and risk of cardiovascular disease. Ann Intern Med. (1976) 85:447–52. doi: 10.7326/0003-4819-85-4-447 970770

[34] Carr MC . The emergence of the metabolic syndrome with menopause. J Clin Endocrinol Metab. (2003) 88:2404–11. doi: 10.1210/jc.2003-030242 12788835

[35] Mauvais-Jarvis F . Sex differences in metabolic homeostasis, diabetes, and obesity. Biol Sex Differ. (2015) 6:14. doi: 10.1186/s13293-015-0033-y 26339468 PMC4559072

[36] Tramunt B Smati S Grandgeorge N Lenfant F Arnal JF Montagner A . Sex differences in metabolic regulation and diabetes susceptibility. Diabetologia. (2020) 63:453–61. doi: 10.1007/s00125-019-05040-3 31754750 PMC6997275

[37] American Diabetes Association Professional Practice Committee . 2. Diagnosis and classification of diabetes: standards of care in diabetes—2024. Diabetes Care. (2023) 47:S20–42. doi: 10.2337/dc24-S002 38078589 PMC10725812

[38] Rolla M Jawiarczyk-Przybyłowska A Halupczok-Żyła J Kałużny M Konopka BM Błoniecka I . Complications and comorbidities of acromegaly—retrospective study in Polish center. Front Endocrinol. (2021) 12. doi: 10.3389/fendo.2021.642131 33796075 PMC8009182

[39] Pivonello R Auriemma RS Grasso LF Pivonello C Simeoli C Patalano R . Complications of acromegaly: cardiovascular, respiratory and metabolic comorbidities. Pituitary. (2017) 20:46–62. doi: 10.1007/s11102-017-0797-7 28224405

[40] Abreu Lomba A Patiño Arboleda M Carvajal Ortiz R Buitrago Gomez N Pinzón Tovar A Vernaza Trujillo DA . Clinical and treatment response differences according to age and gender in patients with acromegaly in Colombia: a retrospective study. Rev Colomb Endocrinol Diabetes Metab. (2025) 12:30–50. doi: 10.53853/encr.12.1.904

